# Rodent mothers increase vigilance behaviour when facing infanticide risk

**DOI:** 10.1038/s41598-019-48459-9

**Published:** 2019-08-19

**Authors:** Merel C. Breedveld, Remco Folkertsma, Jana A. Eccard

**Affiliations:** 1 Animal Ecology, Institute of Biochemistry and Biology, University of Potsdam, Potsdam, Germany; 2 Evolutionary Adaptive Genomics, Institute of Biochemistry and Biology, University of Potsdam, Potsdam, Germany

**Keywords:** Evolutionary theory, Behavioural ecology

## Abstract

Infanticide, the killing of unrelated young, is widespread and frequently driven by sexual conflict. Especially in mammals with exclusive maternal care, infanticide by males is common and females suffer fitness costs. Recognizing infanticide risk and adjusting offspring protection accordingly should therefore be adaptive in female mammals. Using a small mammal (*Myodes glareolus*) in outdoor enclosures, we investigated whether lactating mothers adjust offspring protection, and potential mate search behaviour, in response to different infanticide risk levels. We presented the scent of the litter’s sire or of a stranger male near the female’s nest, and observed female nest presence and movement by radiotracking. While both scents simulated a mating opportunity, they represented lower (sire) and higher (stranger) infanticide risk. Compared to the sire treatment, females in the stranger treatment left their nest more often, showed increased activity and stayed closer to the nest, suggesting offspring protection from outside the nest through elevated alertness and vigilance. Females with larger litters spent more time investigating scents and used more space in the sire but not in the stranger treatment. Thus, current investment size affected odour inspection and resource acquisition under higher risk. Adjusting nest protection and resource acquisition to infanticide risk could allow mothers to elicit appropriate (fitness-saving) counterstrategies, and thus, may be widespread.

## Introduction

The killing of conspecific young by males, i.e. infanticide^1,2^, is a widespread phenomenon that occurs throughout the animal kingdom^2,3^. It is especially common in mammalian species with altricial young^4^, including primates^5^, carnivores^6,7^, and rodents^8–11^. Infanticide can provide the perpetrator an increased fitness by advancing reproductive access to mothers, thus producing a sexual conflict^4^. Alternatively, infanticide may provide a direct nutritional gain, or, through decreased competition, a gain in access to limited resources (e.g. food or space) for the perpetrator and/or its offspring^1,11^. What ever the adaptive significance of infanticide for males may be, immense fitness costs are paid by the targeted female. Therefore, infanticide is an extreme form of sexual conflict and predicted to be an important driver of individual behavioural adaptations^4,12^. In fact, it has led to the evolution of a wide range of female counterstrategies to protect offspring^4^, including territoriality, vigilance behaviour and aggression against intruders, sexual promiscuity to confuse paternity, and mate choice in favour of dominant (i.e. high ranking and often more prone to commit infanticide) males^12–15^.

The time and energy spent on protecting offspring cannot at the same time be invested in other essential tasks, including foraging and reproduction, e.g. investigating potential mating partners for future matings. In order to balance investment between these essential tasks, the energy spent on offspring protection should be adjusted to the existing risk level^16^. Adjusting behaviour to infanticide risk depends on the ability to recognise risk levels, and may be especially important in species facing a high risk, such as species with concurrent lactation and pregnancy^17^. Here, females must mate while caring for existing young to achieve maximum fitness, and thus face pronounced trade-offs between protection of current offspring and reproduction of future offspring. This is the case in rodents. Not only do female rodents themselves face an increased risk of predation during mating^18^, in addition, the nestling litter (i.e. the current reproductive investment) they are temporarily not protecting while mating face an increased risk of infanticide. However, staying with the litter to protect it from infanticide could mean missing out on a potential mate (i.e. investment in a future litter). Moreover, staying with the litter for mating could attract potentially infanticidal males to the nest, and may lead to females having to use costly maternal aggression for offspring protection^15^. Encountering mate partners outside the nest could provide a less risky mating strategy with indirect offspring protection benefits. In summary, female rodents may have evolved the ability to recognise infanticide risk in order to optimally adjust behaviour. Since rodents communicate mainly through olfactory signals^19,20^, and odours from familiar individuals and/or relatives commonly elicit different behavioural responses than odours from unfamiliar/unrelated individuals^21,22^, recognition of infanticide risk may occur through conspecific odour cues, i.e. social odours.

To determine whether infanticide risk leads to adjustments in behaviour driven by fundamental trade-offs between investment in current and future reproduction, we studied the nest defence and explorative behaviour, e.g. for acquiring mates or food resources, of lactating, non-pregnant rodent females exposed to the scent of males (potential mates) presenting differing levels of infanticide risk. We used bank vole, *Myodes glareolus*, mothers in large spatial outdoor enclosures, and studied the behavioural response to the presence of the scent of a male posing either a lower or a higher infanticide risk, as well as their behaviour during a scentless control. In the sire treatment, females received the scent of the familiar sire of their current litter, which poses a lower risk to the offspring, while in the stranger treatment, females received the scent of an unfamiliar male, which poses a higher infanticide risk in microtine rodents^17,23^. Specifically, we investigated whether 1) females flexibly adjust their nest protection behaviour in response to the infanticide risk level, and whether 2) females leave their nest to actively investigate the male scent to obtain information about the donor male, e.g. out of mating interest and/or for recognition of infanticide risk. Since behavioural plasticity can be costly^24^, the ability to recognise infanticide risk and flexibly adjust reproductive behaviour in response to varying risk levels may only be beneficial in species where infanticide acts as a strong selective force. Moreover, showing flexible responses to risk is predicted to pay off only when the risk level is not constant (this was shown for predation risk^25^ and should be similar for infanticide risk^26^). In line with these conditions, infanticide may act as a strong selective force in the bank vole, since it is common and has a large effect on reproductive success and the recruitment of young into populations^10,11,27^. Furthermore, temporal variation in infanticide risk exists, resulting from the overlap of home ranges between males and females and male visits to female territories^28^. Thus, bank vole females may indeed respond plastically when recognising changes in infanticide risk, and may increase allocation to offspring protection under temporarily elevated risk levels^25^.

We predicted that females should recognise different levels of infanticide risk posed by conspecifics in their territory, i.e. potential intruders, and should show behavioural changes in response to the perceived risk for their nestling litter. Specifically, we predicted that under a higher infanticide risk females will increase investment in current reproduction to avoid losing offspring, for instance, by staying in or near the nest, by increasing vigilance behaviour, or by deterring potential intruders. In the treatment simulating a lower infanticide risk, we predicted females to invest less time in offspring protection, and, potentially, more time in future reproduction, for instance, by leaving their litter more often to encounter a mating partner. Alternatively, if females do not recognise the level of infanticide risk, we predicted to find no differences in female behaviour between treatments. Since a female’s fitness costs of infanticide depend on the value of the current parental investment^29^, we predicted litter size to influence the magnitude of a female’s behavioural response to infanticide risk.

## Results

### Absence from the nest

**Figure 1:**
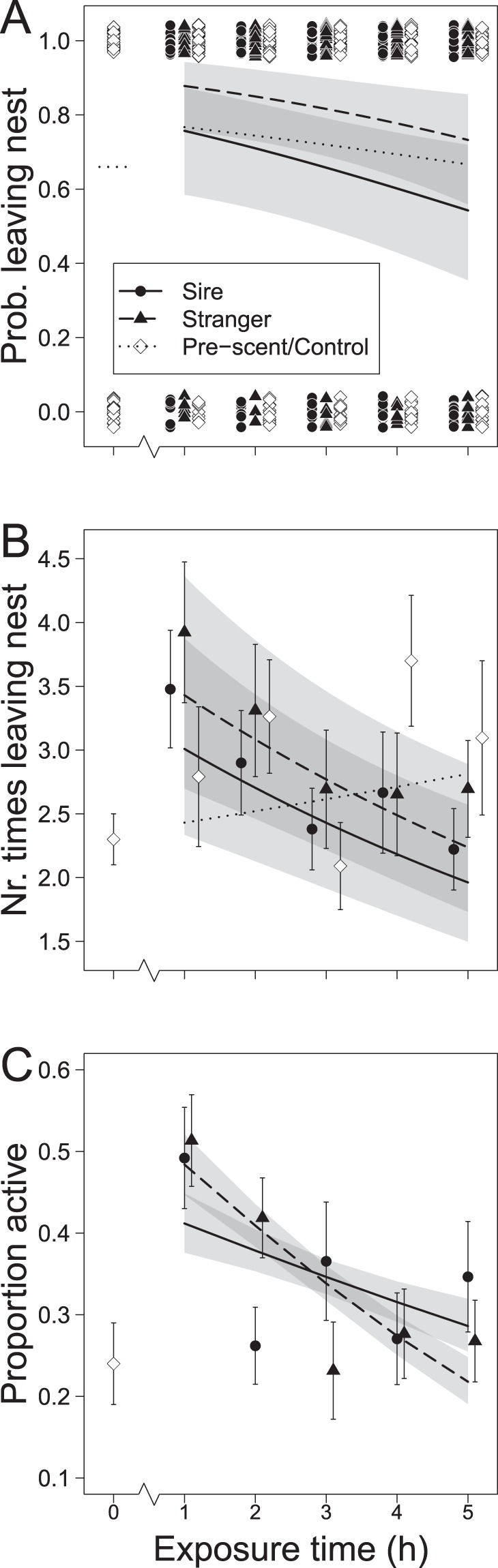
Effects of scent treatment and exposure time on behaviour of 35 lactating, non-pregnant bank vole (*Myodes glareolus*) females, following the spread of the scent of their litter’s sire (Sire; circles), of an unfamiliar male (Stranger; triangles), and of scentless cage bedding (Control; diamonds); and behaviour during the hour prior to spread (exposure time = 0, Pre-scent): (**A**) probability to leave the nest, (**B**) number of times leaving the nest, and (**C**) proportion of time spent active (for a subset of 22 females). Model predictions (lines: Sire continuous, Stranger dashed, Control dotted) and 95% confidence intervals (shaded areas; excluding Control) for time after exposure are shown.

In all but one out of 92 trials (35 bank vole females with three risk levels each; not all trials yielded data), females left their nest 2 to 40 times (mean ± s.e.m. = 10.6 ± 0.8) during the 5 hours following the spread of one of three scent treatments in the enclosure; the scent of their litter’s sire (sire treatment; lower infanticide risk), of an unfamiliar male (stranger treatment; higher infanticide risk), or a scentless control. In the remaining trial, the female did not leave her nest during the 5-hour monitoring period. The hourly probability of leaving the nest was affected by the type of scent treatment (Table 1, Fig. 1A); females exposed to the scent of unfamiliar males (stranger treatment) were more likely to leave the nest (5 h mean: 0.80 ± 0.03) than females from the treatment with sire scent (0.67 ± 0.04; *χ*^2^_*(1)*_ = 8.425, *P* = 0.004) and than females in the scentless control (0.69 ± 0.03; *χ*^2^_*(1)*_ = 2.268, *P* = 0.039), and the latter two groups did not differ (*χ*^2^_*(1)*_ = 0.898, *P* = 0.343). Furthermore, the probability of leaving the nest decreased with hour since applying a scent treatment, i.e. exposure time (estimate ± s.e.m. = −0.19 ± 0.08, Table 1, Fig. 1A). Neither litter size nor any of the interactions explained the probability to leave the nest (all *P* > 0.1).

**Table 1:** Effects of scent treatment (stranger/sire, plus control for RFID data), exposure time, litter size, and interactions on behavioural responses of 35 lactating, non-pregnant bank vole females.

Response variable		RFID data				Telemetry data							
		Prob. leaving (hourly *n* = 92)		N times leaving (hourly *n* = 92)		Activity level (hourly *n* = 41)		Tot. area (95% Kernel, *n* = 36)		Prop. in scent trail (*n* = 36)		Dist. nest (*n* = 1001 locations)	
*Model type*		*GLMM*		*GLMM*		*GLMM*		*LMM*		*GLMM*		*LMM*	
*Family*		*binomial*		*Poisson*		*binomial*		---		*binomial*		---	
*Link/transformation*		*logit*		*logit*		*logit*		*log*		*logit*		*^ 0.5*	
		*χ* ^*2*^_*(d.f.)*_	*P*	*χ* ^*2*^_*(d.f.)*_	*P*	*χ* ^*2*^_*(d.f.)*_	*P*	*χ* ^*2*^_*(d.f.)*_	*P*	*χ* ^*2*^_*(d.f.)*_	*P*	*χ* ^*2*^_*(d.f.)*_	*P*
**Predictor variable**	Scent treatment T	**9.07**_**(2)**_	**0.011**	2.60_**(2)**_	0.273	0.02_(1)_	0.887	1.06_**(1)**_	0.304	2.54_**(1)**_	0.111	**15.33**_**(1)**_	**<0.001**
	Exposure time	**6.08**_**(1)**_	**0.014**	**6.13**_**(1)**_	**0.013**	**88.24**_**(1)**_	**<0.001**	*--- NA ---*		*--- NA ---*		**2.54**_**(1)**_	**0.111**
	Litter size	0.01_**(1)**_	0.928	0.87_**(1)**_	0.351	0.05_**(1)**_	0.817	0.54_**(1)**_	0.462	**4.62**_**(1)**_	**0.031**	0.08_**(1)**_	0.772
	T x Exposure time	0.54_**(2)**_	0.763	*-not converged-*		**13.31**_**(1)**_	**<0.001**	*--- NA ---*		*--- NA ---*		0.80_**(1)**_	0.37
	T x Litter size	0.51_**(2)**_	0.773	*-not converged-*		3.19_**(1)**_	0.074	**7.55**_**(1)**_	**0.006**	2.35_**(1)**_	0.126	0.01_**(1)**_	0.94
*Figure*		*1A*		*1B*		*1C*		*2A*		*2B*		*---*	

The hourly probability of leaving the nest, number of times leaving, and activity level, and the total area visited during the 5-hour monitoring period (log transformed to meet assumption of normality), proportion of locations within the scent trail, and distanced from the nest (square root transformed for normality), were modelled using (generalized) linear mixed models ((G)LMM). Significant effects (alpha level 0.05) are depicted in bold.

The 5 h mean probability of leaving during the stranger treatment was higher than the probability that a female left her nest during the hour before any scent treatment was performed (0.66 ± 0.02; One-sample t-test, *t* = 4.60, *d.f*. = 4, *P* = 0.005; Fig. 1A, pre-scent, hour 0), which was not the case for the sire treatment (*t* = 0.15, *d.f*. = 4, *P* = 0.444) or the control (*t* = 1.09, *d.f*. = 4, *P* = 0.169).

Post-hoc contrasts, performed to discern between behaviour driven by mating interest and nest protection (see Statistical analysis), showed that the probability that females left their nest was not affected by whether or not a male scent was present *per se*: the mean probability that a female left the nest during any scent treatment did not differ from the leaving probability during the scentless control (i.e. sire + stranger scent vs. scentless control, *χ*^2^_*(1)*_ = 0.47, *P*_*adj*_ = 0.494; mean ± s.e.m. during the presence of any male scent: 0.74 ± 0.03). On the other hand, the probability that females left their nest was affected by whether of not infanticide risk was present: the probability of leaving during the risky treatment was higher than the mean leaving probability during any of the two safer treatments (i.e. stranger scent vs. sire scent + scentless control: *χ*^2^_*(1)*_ = 12.4, *P*_*adj*_ < 0.001; mean ± s.e.m. during any safe treatment: 0.68 ± 0.02). Additional post-hoc tests, performed to distinguish behaviour driven by exposure to a male scent from intrinsic temporal differences in activity levels, showed that the probability of leaving the nest was not explained by exposure time during the scentless control, i.e. following the spread of clean cage bedding (i.e. within the control treatment, estimate ± s.e.m. = −0.12 ± 0.13, *χ*^2^_*(1)*_ = 0.9, *P*_*adj*_ = 0.695), while it decreased with exposure time to any male scent (i.e. within sire + stranger treatments, −0.23 ± 0.10, *χ*^2^_*(1)*_ = 5.7, *P*_*adj*_ = 0.034).

The number of times a female left the nest did not depend on the scent type or litter size, but declined with exposure time (estimate ± s.e.m. = −0.06 ± 0.02, Table 1, Fig. 1B). Post-hoc testing revealed that the number of times females left the nest was not affected by exposure time within the control treatment (0.04 ± 0.04, *χ*^2^_*(1)*_ = 0.84, *P*_*adj*_ = 0.717), while it decreased with exposure time to any male scent (−0.11 ± 0.03, *χ*^2^_*(1)*_ = 14.43, *P*_*adj*_ < 0.001).

### Activity level

In the 41 scent trials with radio telemetry data, the total proportion of time during which a female was active (see Behavioural measures) ranged from 0.19 to 0.50 during the first 5 h of exposure (mean proportion in activity = 0.34 ± 0.01), and the proportion active per hour was affected by an interaction between treatment and exposure time (Table 1, Fig. 1C). Post-hoc comparisons showed that while in both the sire and the stranger treatment a female’s proportion of activity per hour reduced with exposure time (sire: estimate ± s.e.m. = −0.1 ± 0.03, *χ*^2^_*(1)*_ = 20.54, *P*_*adj*_ < 0.001; stranger: −0.3 ± 0.03, *χ*^2^_*(1)*_ = 92.78, *P*_*adj*_ < 0.001), the reduction to pre-scent levels occurred later (and was steeper) in the stranger treatment than in the sire treatment (Fig. 1C). Thus, activity levels were highest shortly after a scent was spread and the increased activity levels lasted longer in the stranger treatment. Litter size and its interaction with treatment were not explaining the variable (Table 1).

### Space use

**Figure 2:**
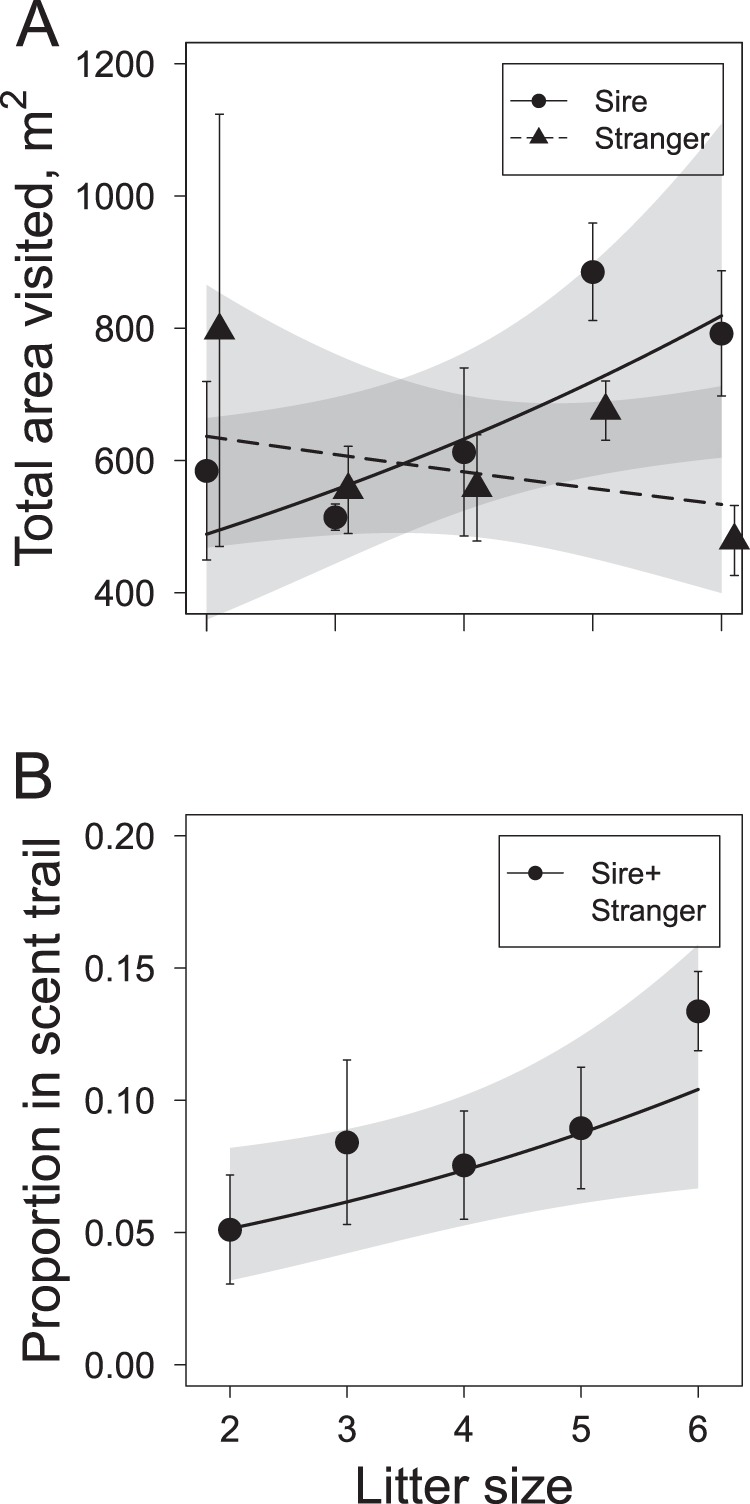
The total area (m^2^) visited by female bank voles (**A**) and the proportion of locations spent in the scent trail (**B**) in relation to litter size during five hours following the spread of the male’s scent. Shown are means (symbols and error bars), model predictions (lines) and 95% confidence intervals (shaded areas).

Females (n = 36) visited a total area of 280 to 1450 m^2^ (95% kernel estimators) during the entire 5-hour monitoring period (640 ± 40 m^2^). Total area visited was affected by an interaction between treatment and litter size (Table 1, Fig. 2A). Post-hoc comparisons showed that the total area visited increased with the size of a female’s litter in the sire treatment (estimate ± s.e.m. = 0.18 ± 0.01, *χ*^2^_*(1)*_ = 11.24, *P*_*adj*_ = 0.002), but not in the stranger treatment (−0.08 ± 0.03, *χ*^2^_*(1)*_ = 3.58, *P*_*adj*_ = 0.117). Exposure time was not important for female space use and was therefore depreciated from these models (see methods).

The proportion of locations within the 60 m^2^ area of the scent trail ranged from 0 to 0.26 (mean = 0.09 ± 0.01), and was larger in females with larger litter sizes (estimate ± s.e.m. = 0.19 ± 0.09, Fig. 2B). Treatment did not explain variation in this proportion (Table 1). The proportion of locations within the scent trail was not affected by litter age, a proxy for female mating interest (*χ*^2^_*(1)*_ = 0.74, *P* = 0.390).

### Distance from the nest

While females were absent from their nest (confirmed by RFID), their distance to the nest ranged from 0 to 29 m (mean = 12 ± 0 m, n = 1001 distances). Females from the stranger treatment stayed closer to the nest (11 ± 0.3 m) than females from the sire treatment (13 ± 0.3 m, Table 1). Thus, the size of area a female used around the nest was 28% smaller in the stranger treatment compared to the sire treatment, based on these two radii around the nest. Litter size, exposure time, and the interactions with treatment did not explain distance to the nest (all *P* > 0.1).

## Discussion

Using an outdoor experimental approach mimicking natural conditions, we showed that females adjust behaviour related to nest-defence and odour investigation, in response to perceiving the scent of either their litter’s sire or of potentially infanticidal, unfamiliar males. Rodent females can recognise previous mates^30^, and males are less likely to kill their own litter, due to familiarisation to the female around mating^31^. Our results suggest that females were able to assess the risk level of infanticide through male odours, and thus fine-tune their behaviour accordingly^32,33^ (but see^34^). This response capacity may allow females to optimize investment in current offspring protection, while saving time and energy to spend on alternative activities, e.g. investing in future reproduction, when infanticide risk is low.

Contrary to our expectations, females did not stay in the nest for offspring protection, as they were more likely to leave their nest when they could perceive a higher infanticide risk (stranger treatment) than when perceiving a lower risk (sire treatment). Staying with the offspring may actually not be a mother’s optimum counterstrategy when perceiving a direct risk of infanticide in their environment. In fact, staying could attract infanticidal intruders to the nest, and a previous study by Ylönen and Horne in a laboratory setting showed that intruders are aggressively attacked by mother bank voles^15^. While maternal aggression was effective to prevent infanticide in that study (contrary to other rodents^32,33,35–37^), it is energetically costly and risky, and could lead to injuries or even death. Thus, aggressive nest defence may be a female’s last resort and would be avoided in a more natural setting. A less costly or less dangerous response may be to find the potential intruder before it actually intrudes. Moreover, females in the aforementioned study acted aggressively towards any intruder physically present at the nest, independent of their infanticidal potential^15^. This indiscriminate aggression may have resulted (in part) from space limits that animals experience in such a laboratory set-up. Females confronted with an intruder directly at their nest may feel so threatened that they are induced to aggressively protect their pups regardless of male type. Therefore, fine-tuned reactions of mothers to infanticide risk levels, as found in the current study, are likely to remain concealed.

The probability and number of times females left their nest decreased with time since a male scent was spread in their territory. This was not the case in the scentless control, confirming male scent as the main driver of changes in female behaviour. With time, the concentration of a volatile odour cue (e.g. a scent mark) decreases, and with it female behavioural responses, highlighting the finesse of olfactory communication^20^. Likewise, activity levels, a proxy for alertness and/or vigilance, were highest shortly after male scent was spread, indicating that females were actively gathering information conveyed by the scent^19^, and/or searching for the “messenger”. Activity levels remained heightened for a longer time in the stranger treatment than in the sire treatment, suggesting that females use both age of the odour cue and risk level to adjust nest protection.

Females facing a higher infanticide risk concentrated their (increased) activity to reduced spatial areas. Specifically, litter size increased the total area that females visited under low infanticide risk (sire treatment; in line with a previous study^38^), but not under high infanticide risk (stranger treatment). Litter size-dependent space use is unsurprising, as larger litters have higher energetic demands and, thus, require a higher resource acquisition by mothers^39^, which can be achieved by an increased food search area. The fact that this association disappeared in the stranger treatment indicates reduced mobility in females perceiving a higher infanticide risk, a typical anti-predatory behaviour reducing resource acquisition^40^. Similarly, the presence of shrews, which can prey on bank vole pups, was shown to decrease home range size and increase time outside the nest of lactating bank vole mothers^41^. Under a sudden high risk for nest predation, resource collection may no longer be a female’s prime activity, and the importance of nest protection increases. In other words, females trade food search for safety of pups, i.e. increase investment in current reproduction. Moreover, reduced space use should decrease the probability of encountering mates^18^, indicating a decreased investment in future reproduction.

Litter size also determined how much time females spent in the male scent trail, potentially obtaining information about the potential intruder through its odour. As females with larger litters have more to lose, the time spent on acquiring information about intruders thus appears to increase with the size of the investment they are currently protecting. This is in accordance with parental investment theory, which predicts that the reproductive value of a litter, and hence optimal parental investment, increases with litter size^29^, and in line with previous findings of bank vole females increasing their nest defence activity with the number of pups in their litter^42^. Time in the scent trail did not seem to result from female mating interest, since it was not affected by the age of a female’s litter; a proxy for her stage in the oestrous cycle (which was unknown directly).

Finally, when mothers were outside of their nest, their distance from the nest was lower under high than under low infanticide risk, in accordance with previous findings in the laboratory^43^ and observed responses to shrews^41^. Altogether, a higher perceived infanticide risk drove lactating females to be more active and leave the nest more often, but to remain closer to it. Mothers must go outside, leaving pups unprotected, from time to time, and time outside may be spent on feeding (i.e. resource acquisition), mating behaviour, and vigilance (i.e. nest protection). Here, females reduced their space use, indicating reduced resource acquisition. Meanwhile, the type of male scent, i.e. the level of infanticide risk, elicited larger behavioural changes than the presence of any male scent per se, i.e. the perceived mate availability. Therefore, we believe that the changes in female behaviour reflect a nest protection strategy, i.e. protection from outside, and not mating interest or resource acquisition.

Maternal aggression, although potentially effective against infanticide (lab observations^15^), may be a last resort, as it can lead to injuries and reduced survival^12^. Aggression could be avoided by females if, through vigilance, the potential intruder is found outside of the nest. Less aggressive counterstrategies may deter the male, such as distracting him away from the nest or mating. Mating with the potentially threatening male is in line with multiple mating as a counterstrategy for infanticide^13^ and previous suggestions that infanticide drives female promiscuity in bank voles^44^. In order to decrease infanticide risk, females may confuse paternity by copulating with multiple males, and female promiscuity should increase with the risk level of infanticide, under the condition that infanticidal males are less likely to kill the offspring of previous sexual partners^1^. Even when females are not in oestrus, i.e. conception is impossible, females may mate with a male to confuse paternity (e.g. in rodents^45^; other mammals^2^). Mating as an infanticide counterstrategy may be the optimal strategy for females that can invest simultaneously in current and future reproduction, such as bank vole females. To conceive their next litter, females must associate with at least one (potentially infanticidal) male while caring for vulnerable pups. As males may simultaneously present an infanticide risk and a mating opportunity, mating could be beneficial to females not only by reducing the chance of infanticide, but by assuring fertilisation at the same time.

In summary, females could recognise the level of infanticide risk posed to their offspring through scent, and fine-tuned their behavioural counterstrategy in response. The observed behavioural changes indicate that females increase their investment in offspring protection, i.e. current reproduction, when perceiving a higher infanticide risk, though vigilance of the nest surroundings. We hypothesise that this strategy may allow females to detect potentially infanticidal intruders early, thereby being one step ahead of them and avoiding the risk of an actual intrusion, and hence of having to use costly aggression. Infanticide is common across the animal kingdom and extremely costly to the victim parent. Therefore, the ability to assess the infanticidal potential of conspecific intruders and to fine-tune parental effort on offspring protection should be adaptive and widespread female traits.

## Materials and Methods

### Species

Bank voles have a promiscuous mating system, with 30% of litters multiply sired in wild populations^46^. Males provide no parental care^47^. Multiple male mating to confuse paternity is one proposed strategy of rodent females against infanticide, as males reduce infanticidal behaviour towards pups that they (potentially) sired^17,23,48^. Experimental evidence suggests that rodent females can recognise the sire of their offspring during lactation^30^ and prefer the presence of familiar males^49^ over unfamiliar males. Other counterstrategies to infanticide may include aggressive nest defence by females against intruders^15^, which may be dangerous and has been observed only in space reduced laboratory set-ups, or preventive terminations of pregnancies (Bruce effect^3,50,51^).

Nest mortality of very young pups through infanticide is common in bank voles, especially by male, but sometimes also by female, perpetrators^10,15,52^. As in many other rodents, males that have mated with and had close contact with a litter’s mother previous to its birth, are less likely to attack the litter^31,53,54^, a strategy that may reduce the probability that a male kills its own offspring. Thus, sires pose a lower infanticide threat to their own offspring, while unfamiliar males pose a higher threat to a female’s offspring^17,23,48^. When a female’s pups have been killed, her next litter’s date of birth is advanced^55^ and its size may be larger^56^, benefitting the perpetrator if he also mates with the female. Infanticide can affect population growth, especially at low densities^11^.

As many rodents, bank voles are seasonal breeders, and females have multiple oestrous cycles during which they become receptive (i.e. are in oestrus) and mating occurs, the latter inducing ovulation^57^. Females are receptive either during post partum oestrus (PPE) or during cycling oestrus (CE)^17,58^. PPE occurs up to 36 h following parturition (i.e. time of oestrus is related to litter age)^55^, and usually lasts shorter than CE, which has a cycle length of 4–5 days^59^. CE commonly occurs when females have not yet mated (at all or in the current season) or when previous mating was unsuccessful, and may be triggered by male odour or presence^60–62^. CE can also occur during lactation^51,63^ (but^55^, see^63^ for discussion). The overlap of reproductive cycles leads to concurrent lactation and gestation^17^, and therefore to a direct trade-off between nursing or nest defence, and behaviour aimed at acquiring (information about potential) copulations, which could importantly determine female mate search strategies. Even though it is often assumed that roaming males visit females for mating in rodents with altricial young^28,64,65^, anecdotal evidence suggest that females actively search for mating partners outside of their nest^66^.

### Pre-Experimental Conditions

During 2012, 2013 and 2014, in total 62 adult bank voles were captured around Potsdam, Germany, using baited live traps (Ugglan special No2, Grahnab, Sweden; with shrew exits^67^. Animals were housed individually in standard makrolon cages (Ehret GmbH, Germany, Typ III: 42 cm × 27 cm × 16 cm), containing bedding, shelter, water and food pellets (ssniff V1594 R/M-H Ered II, Germany) ad libitum. The animal colony was maintained at room temperature (18–23 °C) and natural seasonal photoperiod.

Prior to the experiment, females obtained an RFID chip (Trovan, ISO 100, subcutaneous injection), and a randomly selected male was introduced into each female’s cage and removed after two weeks. Once a female was categorised as gravid, a wooden nest box was introduced in the cage, and the female monitored until parturition. Two to three days after parturition, experimental females received a radio transmitter (Biotrack PIP2, 1.1 g, 150–151 MHz mounted on a cable tie). At a litter age of 4.1 ± 0.3 days (mean ± SD, range = 0–10) the wooden nest box with females and litters was transferred to the study site. Males that sired a litter were kept in a separate cage to obtain cage bedding carrying the scent of the male to be used later in the scent treatments.

To recover females and pups from the enclosure after the experiment, we waited until they were nursing in the nest box (confirmed with radiotelemetry) and collected the box with animals inside. We removed radio transmitters, and kept females with litters until a weaning age of 18–20 days. Following the experiments, females were investigated for animal personality traits (not used in the data presented here).

### Experiment

Thirty-seven lactating non-pregnant females were used in the experiment. These participated in 111 unique trials, performed between July and October of 2012, 2013 and 2014, at the field station of Potsdam University (52°26′18″N 13°00′22″E). Each female received each of three treatments: the familiar scent of the sire of their current litter, to simulate a lower infanticide risk, the scent of an unfamiliar male, to simulate a higher infanticide risk, and a scentless control, reflecting baseline exploration/food-search behaviour. Technical problems and bad weather meant that sometimes data could not be successfully collected during one or more treatments, so that sample sizes differ among analyses; for the final total number of trials per data type see “Behavioural Measures” below.

Nest boxes of two females, each with her litter, were positioned at diagonally opposite corners of a 50 m × 50 m outdoor enclosure, with ca. 42 m distance between them. RFID readers below the nests confirmed that only the mothers entered their nests, and never the other female. Enclosures contained natural herbaceous vegetation, and were surrounded by an escape-proof galvanised metal wall. Fencing surrounding all enclosures provided protection from natural ground predators, while avian predation was not restricted. Our outdoor natural set-up provided a realistic representation of female behaviour in time and space, allowing us to disentangle female behaviour at the nest and her time away from the nest.

The scentless control treatment was performed on day 0, and scent treatments were performed on day 1 and 2 in randomised order. For the control treatment, one litre of (unused) cage bedding was distributed along both scent trails: a straight path of ca. 30 m long and 2 m wide, leading away from each nest and starting at ca. 1 m around the nest. For the scent treatments cage bedding for both scent trails was taken from the cage of the sire of one of the two females’ litter. With this design, one female received the scent of the sire of her litter on day 1 and the scent of a male unfamiliar to her (i.e. stranger) on day 2, while the other female received the scent of an unfamiliar male first and the scent of the sire subsequently. Scent trails were running parallel and had a distance of ca. 30 m between them, so that females following a scent trail would not meet. All scent and control treatments were performed one hour before sunset, and female behaviour was monitored for the following 5 hours, so that the monitoring interval included dusk and evening hours when bank voles are most active^68^.

### Behavioural Measures

#### Presence and absence from the nest box

An RFID reading station was placed under each nest box logging present RFID tags every 17 seconds on average. Logging intervals >1 min were considered as absence of the female from the nest box. We investigated the probability and duration of absence from the nest and the number of times leaving the nest. RFID data was obtained for 92 scent and control trials (from 35 different females).

#### Location and activity in the enclosure

Each outdoor enclosure contained an automated VHF (very high frequency) radio telemetry (ART) system modified after Kays *et al*.^69^ (see supplement), to determine (during the scent trials only) the locations and activity status of females every 3 minutes with an accuracy of 3 to 8 m, depending on wind and air moisture (see supplement). From the locations, space use parameters (see statistics) and distance from the nest were calculated using the R Package adehabitat^70^. With multiple additional data gaps due to collar drop-offs, battery failures, or unexpected rainfall, we obtained high resolution VHF location data plus activity data for 36 scent trials (from 19 different females), and activity data only for 5 additional scent trials (3 different females) where the locations could not be calculated due to logging failures.

### Statistical analyses

All behavioural variables were analysed using (generalized) linear mixed effects models ((G)LMM), including female identity as a random effect to account for pseudoreplication, technical differences among tracking grids, and inter-individual differences in activity levels^71,72^. In all of the initial models, the hourly behaviour of females was modelled, by summing female responses per hour since exposure to the scent. All initial models included scent treatment (sire scent, stranger scent, or (if available) scentless control) as a fixed factor, exposure time (1 to 5 hr) and litter size (1 to 6 offspring) as covariates, and the interactions between scent treatment and the covariates. When exposure time was not significant, the response variable was recalculated for the complete 5-hour post-scent period, and a new model excluding exposure time was run.

Since parental investment can affect a female’s behaviour^43,73^, and older litters may represent a larger investment, we initially also investigated the age of the litters. As predicted (based on the assumption that all pups under 10 days old are vulnerable to infanticide^10^) no effects were found, so litter age was discarded from subsequent models. Other random factors that were tested initially, but excluded due to insignificance (*P* < 0.05), included: calendar date (accounting for differences in season, vegetation, weather, etc.), year, enclosure, and day time of scent spread. Male identity, included to account for potential effects of dominance or different infanticidal tendencies perceived by the females, and also controlling for external effects of pairs of females tested simultaneously, did not explain differences in female behaviour.

We analysed the following variables (see Table 1 for model details): the probability of leaving the nest at least once in a given hour; the number of times leaving the nest per hour (for females that did indeed leave at least once); activity level, i.e. the proportion per hour that a female was active (by defining activity from the telemetry data, see supplement); total area visited by a female during the 5-hour monitoring period, estimated using a 95% kernel utilization distribution (KUD^74^); a female’s estimated proportion of locations within the scent trail, including the nest box (area: ±60 m^2^); and a female’s distance from the nest (using locations determined by telemetry data when the female’s absence from the nest was confirmed by RFID).

Within the RFID data analyses, post-hoc tests were performed to discern between behaviour driven by mating interest and nest protection. We tested the probability that females left the nest in response to whether or not a male scent was spread by running contrasts between any male scent (i.e. sire + stranger scent) and the scentless control, and in response to whether or not infanticide risk was present by contrasting between any safe trials (i.e. sire + control) and risky (i.e. stranger) scent trials. We predicted that if behaviour was driven by mating interest, we should find larger effects in the comparison of scent vs. scentless control. If instead, behaviour was driven by infanticide risk, we predicted to find the larger effects in the comparison of safe vs. risky. Additionally, as a female’s mating interest may depend on her stage in the oestrous cycle, effects of litter age (as a proxy for oestrus status) could reveal behaviour driven by mating interest. Therefore, an additional binomial GLMM was run with the proportion of locations in the scent trail, i.e. female interest in male odour, with litter age as a covariate. Finally, to determine whether our results were indeed driven by the presence of male scent instead of intrinsic differences in activity levels, we tested the probability of and number of times leaving the nest per hour within the control trials. Since scent is volatile and its concentration wears off with time elapsed, we predicted that if the scent treatment was effective, behaviour should change with time since spread of cage bedding in the scent treatments, but not in the scentless control. If instead, factors other than the scent treatment were responsible for behavioural changes, e.g. individual activity rhythms, we predicted a significant effect of time since spread (disturbance) in the control subset.

All analyses were performed in R 3.5.0^75^. Model selection was always performed using likelihood ratio tests and non-significant terms were backward eliminated (*P* > 0.05). Post-hoc tests were adjusted for repeated testing, and adjusted *P* values (*P*_*adj*_) are reported.

### Ethics statement

Experiments were part of a project approved by the Landesamt für Umwelt, Gesundheit und Verbraucherschutz Brandenburg (reference number V3-2347-44-2011). Animals were captured under permission of the Landesumweltamt Brandenburg (reference number RW-7.1 24.01.01.10), and the experiments were in accordance with all applicable international, national, and/or institutional guidelines for the use of animals.

## Supplementary information

ART system

RFID_NestAbsenceData

Telemetry_ActivityData

Telemetry_DistanceData

Telemetry_LocationData

## Data Availability

Data is available as supplementary material.
