# Supplementary material for: Rodent mothers increase vigilance behaviour when facing infanticide risk
Source: Sci Rep. 2019 Aug 19;9:12054. doi: 10.1038/s41598-019-48459-9 (PMC6700316; doi:10.1038/s41598-019-48459-9)
Supplement: Supplementary file 1 — ART system [file 41598_2019_48459_MOESM1_ESM.pdf]

SUPPLEMENTARY INFORMATION

Manuscript: Rodent mothers increase vigilance behaviour when facing infanticide risk

Authors: M. C. Breedveld, R. Folkertsma, J. A. Eccard

ART system

Each outdoor enclosure contained an automated VHF (very high frequency) radio telemetry (ART) system modified after Kays et al.[1], consisting of 8 four-element Yagi antennae (Winkler-Spezialantennen, Germany), set up in 4 pairs (one pair per enclosure corner) with standardised within pair distance and angle, and connected to an automatic 8 channel receiving unit (ARU; Sparrow systems, USA), which was programmed to repeatedly log the signal strength of a transmitter frequency at each antenna sequentially. We converted the distribution of signal strengths into bearings from each corner, that were used to triangulate the location of each transmitter (i.e. female) at set logging intervals (every 3 ± 0.2 minutes). Based on the locations, space use parameters of females (total area visited, ratio of locations within the scent trail, distance from the nest; see statistics) were calculated using the R Package *adehabitat* [2]. Variations in signal strengths within sequentially logged signals of one frequency on the same antenna were further used to derive the activity status of a female. A higher variation indicates a greater change in transmitter location or transmitter position relative to the static, receiving antennae. A female was defined to be active at a logging interval when the sum of the variation among signal strengths of subsequent signals across all logged antenna channels was above a threshold level (where ± 25 % of all positions were active, based on minimum natural activity levels in bank voles[3]). Further, preliminary analysis revealed, that threshold levels between 15%-40% of logging intervals considered active revealed the same temporal distribution of activity peaks over the day[4]. However, with this method we were not able to compare the overall activity levels among treatments but the distribution of activity over the observation interval. Signal variation can also be produced while the female is moving inside the nest, and an immobile female outside the nest can produce a signal classified as inactive. Therefore, activity determined through VHF telemetry is not congruent to nest presence and absence.

Location estimates had a median deviation of 8.5m (based on true vs. triangulated position of beacons), and a consistency of ± 3.4m of repeated virtual locations from an immobile transmitter. Note that for animals located at the ground, location accuracy is strongly affected by contours of the soil surface, but these differences should not affect the analyses as each female performed all treatments at the same location (repeated measures). Moreover, during the conversion of signal strengths to bearings, sequentially logged signals were smoothed, allowing for further improvement of accuracy of final locations.

References

1. Kays, R. *et al.* Tracking animal location and activity with an automated radio telemetry system in a tropical rainforest. *Comput. J.* **54**, 1931–1948 (2011).
2. Calenge, C. Analysis of animal movements in R: the adehabitatLT package. *Office national de la chasse et de la faune sauvage* 1–82 (2015).
3. Mironov, A. D. Spatial and temporal organization of populations of the bank vole, Clethrionomys glareolus. in *Social systems and population cycles in voles* (eds. Tamarin, R. H., Ostfeld, R. S., Pugh, S. R. & Bujalska, G.) 181–192 (Birkhäuser Verslag, 1990).
4. Hoffmann, J., Palme, R. & Eccard, J. A. Long-term dim light during nighttime changes activity patterns and space use in experimental small mammal populations. *Environ. Pollut.* **238**, 844–851 (2018).
